# Paroxysmal Sustained Ventricular Tachycardia with Cardiac Arrest and Myocardial Infarction in 29-Year-Old Man Addicted to Medical Marijuana—It Never Rains but It Pours

**DOI:** 10.3390/healthcare10102024

**Published:** 2022-10-13

**Authors:** Jerzy Wiliński, Anna Skwarek, Iwona Chrzan, Aleksander Zeliaś, Radosław Borek, Dominika Elżbieta Dykla, Maria Bober-Fotopoulos, Dariusz Dudek

**Affiliations:** 1 Center for Invasive Cardiology, Electrotherapy and Angiology, 33-300 Nowy Sącz, Poland; 2 Department of Internal Medicine with Cardiology Subdivision, Blessed Marta Wiecka District Hospital, 32-700 Bochnia, Poland; 3 2nd Department of Cardiology, Jagiellonian University Medical College, 31-501 Krakow, Poland

**Keywords:** marijuana, ventricular tachycardia, myocardial infarction, cardiovascular magnetic resonance, heart

## Abstract

This article presents the case of a 29-year-old male patient, addicted to prescribed medical marijuana administered for mixed anxiety and depressive disorder and without classic cardiovascular risk factors and history of myocarditis, suffering from episodes of paroxysmal hemodynamically unstable ventricular tachycardia. Cardiovascular magnetic resonance imaging of the heart revealed disseminated non-ischemic myocardial injury lesions of subepicardial and intramuscular location. Additionally, the individual experienced myocardial infarction without ST segment elevation following marijuana intake. Treatment required implantation of a cardioverter-defibrillator and ablation of the myocardial areas responsible for the origin of the arrhythmia, as well as appropriate pharmacotherapy and marijuana addiction treatment.

## 1. Introduction

Marijuana—dried leaves obtained from plants of a type of hemp (Cannabis)—is an increasingly common stimulant used, and not only by young people. As a psychoactive drug it finds more and more medical applications, such as relief of moderate to severe symptoms of spasticity in patients with multiple sclerosis, treating chronic pain, including cancer pain, nausea during chemotherapy, as an anticonvulsant, in the treatment of anorexia, arthritis, glaucoma and migraine, improving appetite in acquired immune deficiency syndrome and sleep quality, and relieving tics in Tourrette’s syndrome [1]. It should be remembered that the regular use of marijuana might have side effects. It increases the level of catecholamines, which contributes to increased oxygen use in the heart muscle and exerts pro-thrombotic effects, resulting from the action on cannabinoid receptors on the blood platelets’ surface. The contractility disorders of the myocardium mediated by the CRB1 receptor have also been described. The negative clinical consequences of these biological actions include cardiac arrhythmias: supraventricular, also atrial fibrillation, and ventricular of various severity; increased risk of myocardial infarction, including ones complicated by cardiogenic shock, and the development of dilated cardiomyopathy also in young individuals, stroke, vasculitis and postural hypotension [2,3].

Epidemiologic data examining cardiovascular events and arrhythmia prevalence among users of marijuana are lacking. A recent cross-sectional study showed that history of cardiovascular diseases was more prevalent amongst the respondents using marijuana than in non-users (65.42% vs. 52.81%; *p* < 0.0001) [4]. Another cross-sectional study, on associations between marijuana smoking and arrhythmias in 1485 participants using single-use monitors, revealed that participants reporting current use of marijuana (compared with never users) had more premature atrial contractions per hour (adjusted geometric mean ratio [GMR] 1.22, 95% CI, 0.72–2.13) and more episodes of supraventricular tachycardia per day (GMR 1.42, 95% confidence interval [CI], 0.87–2.32). Considering ventricular arrhythmias, they had more runs of non-sustained VT per day (GMR 1.28, 95% CI, 0.95–1.73). Moreover, frequent use of marijuana was associated with more episodes of non-sustained VT per day (GMR 1.56, 95% CI 1.13–2.17) [5].

## 2. Case Presentation

**Figure 1:**
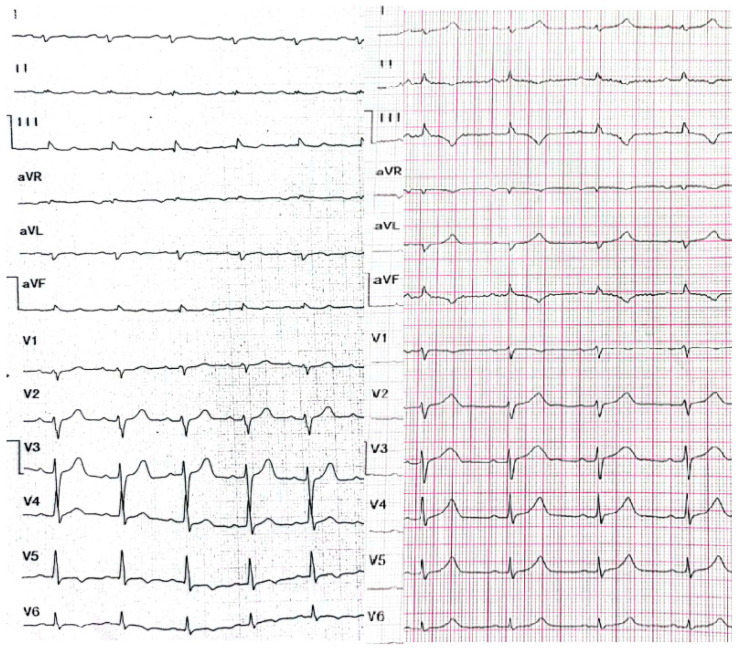
Resting electrocardiograms at standard paper speed of 25 mm/s and normal calibration of 1 mV = 10 mm (description in the text).

**Figure 2:**
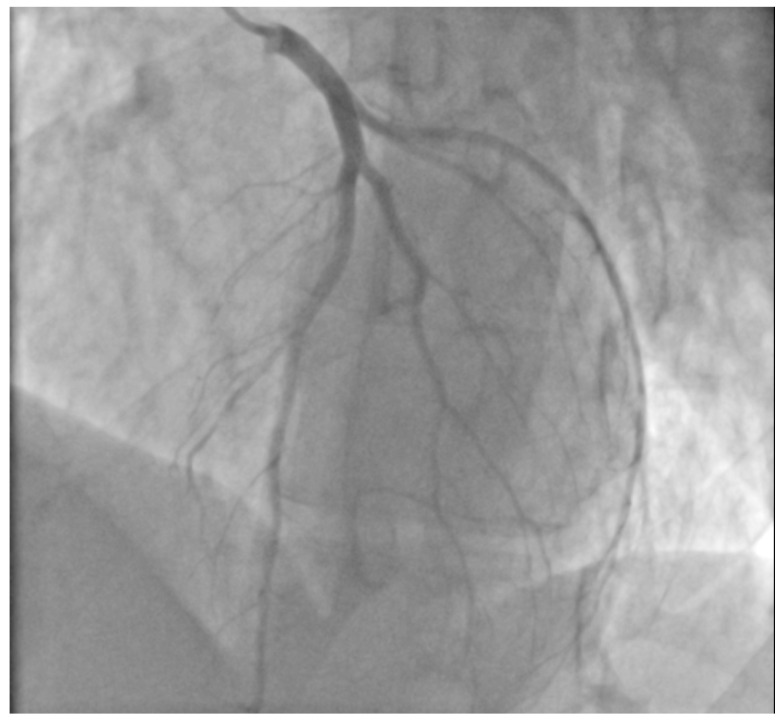
Coronary angiography (LAO cranial projection). There were no changes in the left main coronary artery, the left anterior descending and the circumflex arteries.

**Figure 3:**
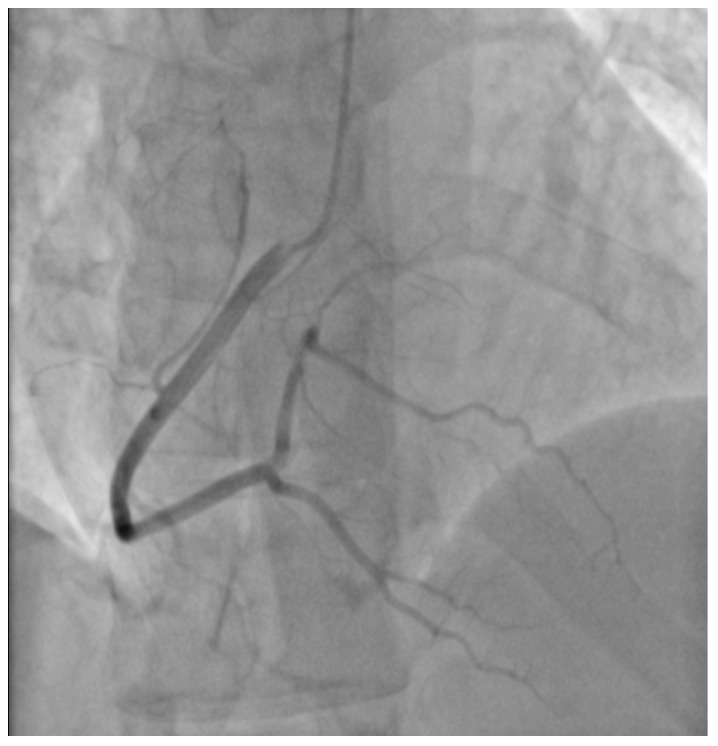
Cronarography (RAO straight projection). No lesions in the right coronary artery.

A 29-year-old male patient, a manual worker without history of overt infectious myocarditis, without classic risk factors for cardiovascular diseases, with mixed anxiety and depressive disorder, was treated with prescribed off-label medical marijuana (delta-9-tetrahydrocannabinol THC 19% and cannabidiol CBD < 1%, bred from Lemon Skunk Cannabis Strain in the form of unprocessed dried leaves) for around 3 years, smoked at least 3–4 times a day and was diagnosed with severe cannabis use disorder (continued use of cannabis despite clinically significant impairment) [6]. It was diagnosed by a psychiatrist according to the fifth version of the *Diagnostic and Statistical Manual of Mental Disorders (DSM-5)* with 8 out of 11 criteria met: hazardous use, social/interpersonal problems, neglected major roles, withdrawal, used larger amounts/longer, much time spent using, activities given up and craving [7]. Moreover, the patient was categorized as a chronic user [8]. He had been suffering from paroxysmal hemodynamically unstable ventricular tachycardia (VT). The first episode occurred 30 months ago and the patient was referred from an emergency room to a distant cardiology center, where upon coronarography, apart from myocardial bridge localized in the middle segment of the left anterior descending coronary artery no lesions in the coronary arteries were recorded. There were no specific abnormalities recognized in resting ECG (Figure 1, left panel). In the echocardiogram the structure and function of the heart was normal, apart from a mildly compromised left ventricular systolic function with left ventricular ejection fraction (LVEF) of 43%. At that time the patient did not consent to the proposed diagnostics of VT consisting of cardiovascular magnetic resonance imaging of the heart, electrophysiological study, genetic testing and therapy with subcutaneous implantable cardioverter-defibrillator. He received metoprolol in extended-release drug formulation (metoprolol succinate) of 25 mg q.d. due to intolerance of higher doses, potassium, magnesium and vitamin B6 supplementation. Within this period, the subject experienced at least six episodes of VT that required medical assistance. Moreover, 7 months earlier he was admitted to the catheterization unit again after an acute episode of retrosternal pain following marijuana intake. Once more, no lesions within the coronary arteries were observed in coronarography (Figure 2 and Figure 3). ECG showed no signs of ischemia, echocardiography was without segmental dysfunction of cardiac muscle, and LVEF equaled 40%. Noteworthy, the top troponin T serum concentration exceeded the upper end of the scale (>10,000 ng/mL), with CPK of 4167 U/L, CK-MB-428 U/L and CRP 3.4 mg/L. Non-ST-elevation myocardial infarction was diagnosed but the patient still declined extended diagnostics and marijuana addiction therapy. The treatment was supplemented with acetylsalicylic acid 75 mg q.d., clopidogrel 75 mg q.d., ramipril 1.25 mg q.d., rosuvastatin 10 mg q.d., eplerenone 25 mg q.d. and pantoprazole 20 mg q.d.

**Figure 4:**
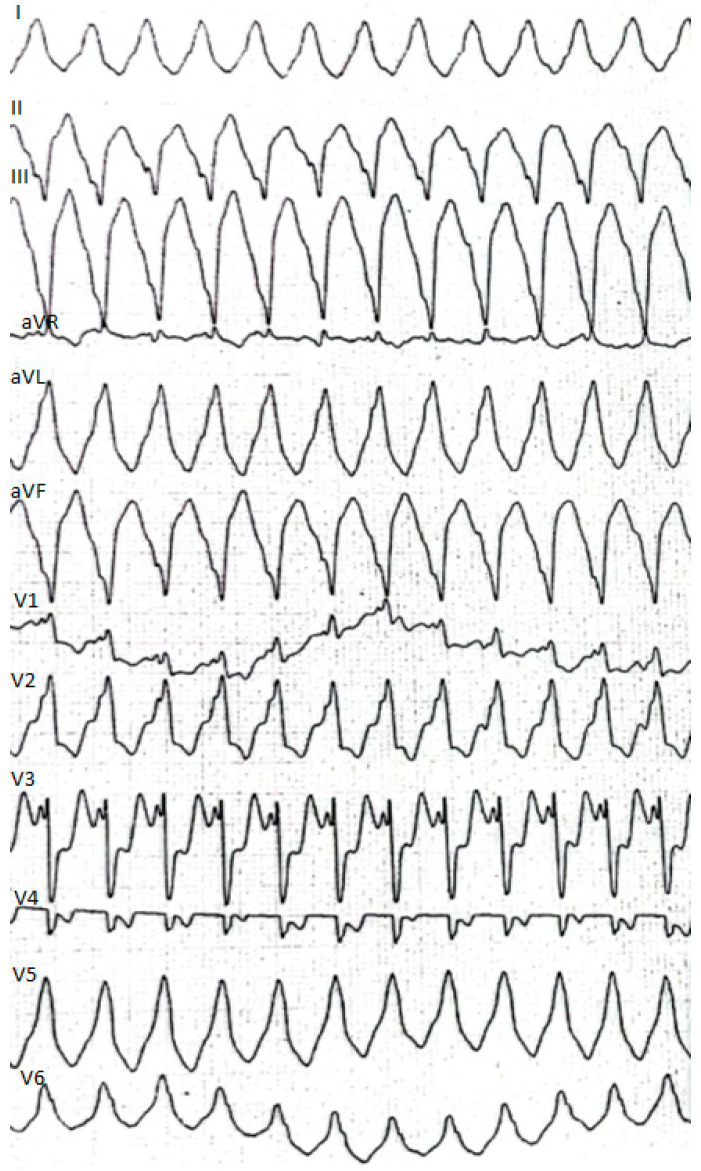
Resting electrocardiogram recording ventricular tachycardia at standard paper speed of 25 mm/s and normal calibration of 1 mV = 10 mm.

This time the individual was admitted to our cardiology center after another episode of VT, with cardiac arrest in the mechanism of VT at an emergency room (VT score—4 points, positive result of the Vereckei, Brugada and limb algorithms for the diagnosis of VT on ECG, Figure 4) treated with electrical cardioversion for further treatment [9,10]. The physical examination showed no significant abnormalities, the patient’s weight was 69 kg, height—190 cm, body mass index–BMI–19.11 kg/m^2^. Peripheral blood saturation—96%, body temperature—36.7 degrees C. Blood pressure was in the range of 100–110/60–70 mm Hg. In laboratory tests: white blood cells count—WBC—8.45 K/µL; hemoglobin—Hgb—13.3 g/dL; platelets count—PLT—260 K/µL; troponin T—317.6 ng/mL (norm < 30); N-terminal pro-brain natriuretic peptide—NT-proBNP—905 pg/mL; creatine phosphokinase—CPK—352 U/L; MB iso-enzyme of creatine kinase-CK-MB-24 U/L (6.8% CPK); thyroid-stimulating hormone—TSH—0.805 µU/mL; total cholesterol—2.6 mmol/L; low-density lipoprotein cholesterol—LDL—1.35 mmol/L; high-density lipoprotein cholesterol—HDL—0.89 mmol/L; triglycerydes—TG—0.87 mmol/L; glucose—5.6 mmol/L; creatinine—77 µmol/L (estimated glomerular filtration rate—eGFR > 90 mL/1.73 m^2^/min); sodium—141 mmol/L; potassium—3.61 mmol/L; chlorides—102 mmol/L; C-reactive protein—CRP—1.0 mg/L; d-dimer—413 ng/mL; alcohol—0.2 per mille; international normalized ratio- INR—1.23; activated partial thromboplastin time—aPTT—34 s; Abbott antigen test for SARS-CoV-2 virus negative; PCR test for COVID-19 also negative. The resting electrocardiogram revealed a sinus rhythm of 76 beats/min, the axis of the heart inclined moderately to the right, low voltage of QRS complexes in the limb leads, negative T waves in V5 and V6, and positive–negative in aVL. PQ equaled 160 ms, QRS—90 ms, QTc—440 ms (Figure 1, right panel). On echocardiography, the left ventricle was not enlarged (its end-diastolic dimension—55 mm), the interventricular septum was moderately thickened (11 mm in the diastolic phase), normal left ventricular systolic function was observed, with no segmental abnormalities of contractility (left ventricular ejection fraction—LVEF was 55%). No other significant abnormalities, including significant valve defects, were observed.

**Figure 5:**
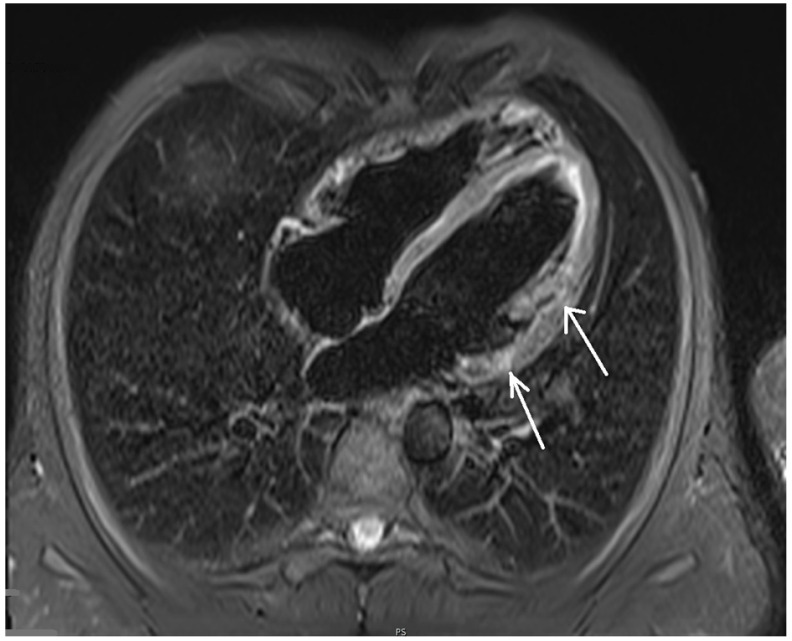
Magnetic resonance imaging of the heart showing disseminated subepicardial and midwall late enhancement lesions here in the lateral wall of the left ventricle (arrows).

In the cardiovascular magnetic resonance imaging of the heart, left and right ventricle sizes were within normal ranges, correct wall thickness, correct global and segmental contractility and normal ejection fractions of both ventricles (58% and 51%, respectively) were observed. After administration of the contrast agent, the areas of delayed epicardial and intramuscular enhancement were visualized, mainly in the area of the inferior and lateral walls of the left ventricle. No signs of myocardial edema and hyperemia, met in acute myocarditis, could be visualized (Figure 5). Those fibrotic lesions formed the picture of nonischemic myocardial damage in the picture of past myocarditis [11,12,13].

**Figure 6:**
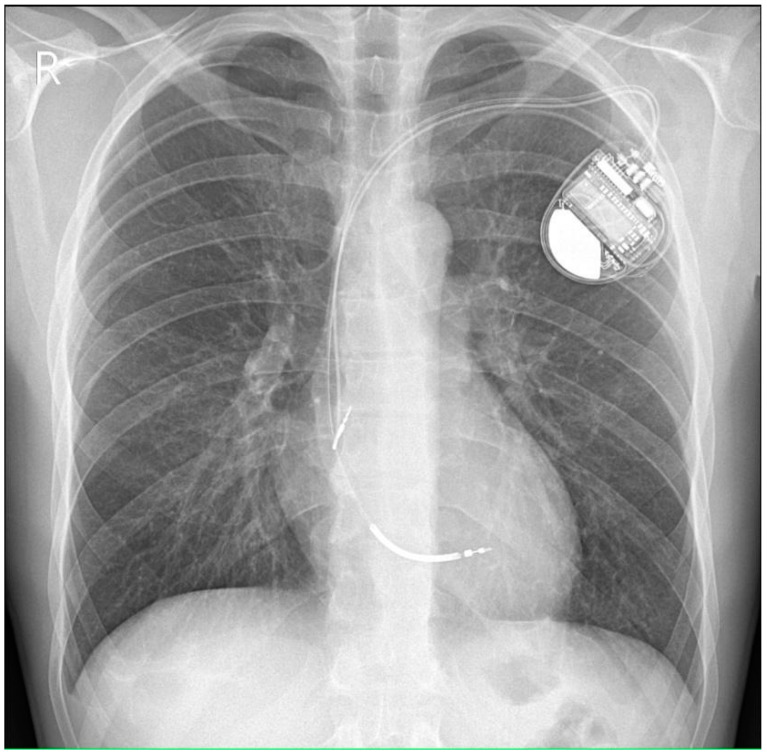
Chest X-ray in the PA projection—condition after implantation of a dual-chamber cardioverter-defibrillator—a single-coil defibrillating electrode with a tip in the middle of the interventricular septum and atrial electrode with a tip in the right atrium appendage. No other abnormalities are seen in the X-ray image.

A dual-chamber cardioverter-defibrillator (ICD DR, he did not consent to have subcutaneous implantable cardioverter defibrillator) was implanted for the secondary prevention of sudden cardiac arrest and life-threatening ventricular arrhythmia (Figure 6). Antiarrhythmic treatment was intensified: the dose of metoprolol succinate was increased from 25 mg to 50 mg q.d.

**Figure 7:**
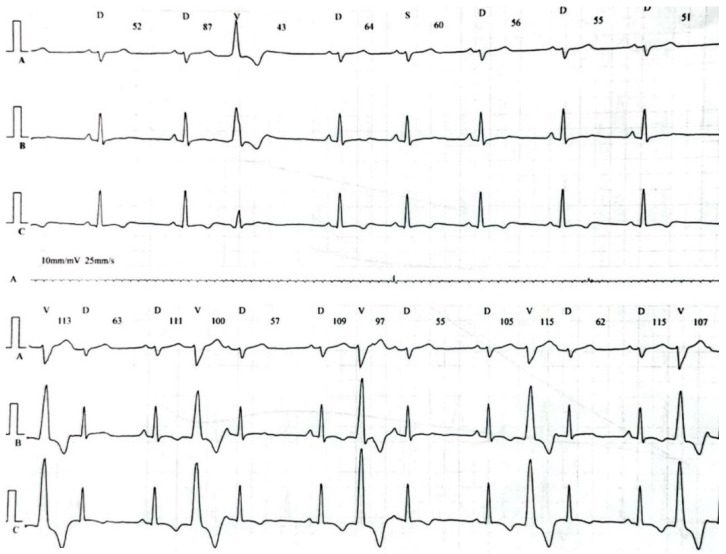
Excerpt from 24-h Holter monitoring of electrocardiogram. Sinus rhythm, additional ventricular beats with 2 different morphologies (single beats on top and concealed beats on bottom panel).

Fourteen days after discharge from the hospital, the patient reported for a follow-up visit. The wound healing after ICD implantation was normal, the parameters of the device (sensing, thresholds for atrial and ventricular stimulation, resistance of the pacing system and the high-energy system) were within the normal range. No complex ventricular arrhythmias were noted in the incorporated ICD ECG monitoring. Pharmacotherapy was maintained. After another 20 days, the patient was admitted to our cardiology center due to an electrical storm with recurrent VT in which some VT episodes were interrupted with antiarrhythmic therapy of the ICD, whereas some required adequate interventions of high-energy shocks. Temporary treatment with i.v. amiodarone and metoprolol tartrate p.o. was introduced and the patient was referred for urgent electrophysiological examination. The patient underwent RF ablation of the areas in the left ventricle within the interventricular septum responsible for the development and maintenance of tachycardia, resulting in the elimination of VT. Pharmacotherapy was maintained in the same way as after ICD implantation, including antiarrhythmic treatment with metoprolol sustained-release 50 mg/day. There was no recurrence of VT during the 3-month follow-up. In the 24-h control Holter ECG monitoring, 260 single ventricular accessory beats, without complex ventricular arrhythmias, were recorded (Figure 7). In addition, during each hospitalization, the patient was instructed about the harmfulness of the use of marijuana and alcohol, and after discharge from the ward he was referred to a mental health clinic to treat anxiety disorders and addiction to psychoactive substances.

## 3. Discussion

Paroxysmal VT in young people is a rare phenomenon, with a spectrum of symptoms ranging from oligosymptomatic to cardiac arrest. The most common form of VT in this patient group is VT without organic heart disease, with an arrhythmia origin in the outflow tract of the right ventricle of a relatively mild course. Less common types of VT include: idiopathic VT of other localizations, VT based on the diseased muscle of the ventricles, also on the background of ischemic heart disease; in channelopathies, cardiomyopathies, in patients with congenital heart disease and after surgical operations for various reasons. A separate group is VT induced by drugs and various stimulants. There are also forms with complex etiology [14]. All these causes need to be considered in the diagnostics of VT, especially in young patients.

The gold standard for noninvasive imaging in heart muscle pathologies—magnetic resonance imaging—has a well-defined typical pattern for several ischemic and non-ischemic disease processes. The diffuse late gadolinium enhancement lesions, located in the lateral, inferolateral or inferior wall with a midwall to subepicardial distribution, without oedema, hyperemia/capillary leakage with irreversible injury of necrosis/fibrosis indicate past myocarditis, not only of infectious background also of toxic myocarditis like a marijuana-related one [6,7,8]. Recent years have yielded multiple cases of cannabis-induced myocarditis varying from asymptomatic cases to end-stage cardiomyopathy and death [15,16,17,18,19,20,21]. Noteworthy, marijuana contains more than 460 active chemical compounds including THC which acts via the CB1 and CB2 receptors that are widely distributed in different tissues of the body. Only in the heart, the heart rate, rhythm, coronary flow, coagulation and cell metabolism are affected. It is not clear if marijuana itself causes myocarditis or if the etiopathogenesis is actually related to the contaminants in marijuana such as pesticides and heavy metals. Moreover, marijuana can exacerbate the already ongoing inflammatory process in the myocardium [13]. Since there are no pathognomonic signs of marijuana induced myocarditis in diagnostic imaging, the diagnosis is commonly made on the basis of the relation of symptoms with exposition to cannabis and excluding other causes. In our patient marijuna was the most probable culprit of myocardial damage.

Another potentially life-threatening complication of marijuana is myocardial infarction. The mechanisms leading to acute myocardial ischemia cover artery vasospasm and dissection, vasculitis, increased platelet aggregation and coronary artery thrombosis, autonomic nervous system imbalance, oxidative stress, endothelial injury and inflammation secondary to impurities and even hypertensive urgency from abrupt withdrawal [22,23,24]. Clinical manifestation varies from angina to cardiac arrest. Young healthy men without cardiovascular risk factors often present with transient (classified as non-ST acute coronary syndromes) or persistent ST-segment elevations, and soared troponin concentrations with no inflammatory markers elevation or findings of myocarditis on MRI (which was observed in the presented case) [25]. Moreover, marijuana has been recognized as a significant contributor to early-onset cardiovascular diseases next to cocaine, amphetamines and alcohol [26,27]. A recent cross-sectional study, using pooled data from the 2017 and 2018 cohorts of the American Behavioral Risk Factor Surveillance System survey of US adults, showed that history of myocardial infarction was more frequent among recent cannabis users relative to nonusers (adjusted odds ratio [OR] 2.07, 95% CI, 1.12–3.82) and was associated with cannabis use of more than four times per month (OR 2.31, 95% CI, 1.18–4.50), and with smoking as a primary method of consumption (adjusted OR 2.01, 95% CI, 1.02–3.98) [28]. In a multi-center study, the risk of myocardial infarction onset was elevated 4.8-fold (95% CI, 2.9–9.5, *p* < 0.001) within the first hour after smoking marijuana [29]. Another crucial factor is that cannabis use is frequently accompanied by tobacco smoking [24]. Importantly, myocardial infarction with non-obstructive coronary arteries (MINOCA) is a disease syndrome with a prognosis at least as serious as an atherosclerotic myocardial infarction. Additionally, the final diagnosis of MINOCA causes may confirm the coronary cause (dissection, spasm, thrombus) or differ significantly from the initial diagnosis (myocarditis, takotsubo cardiomyopathy, use of stimulants) [30,31]. At times, the primary mechanism cannot be identified and documented like in our case.

The described case clearly shows how important the role of complex treatment of ventricular arrhythmias is—excluding reversible causes of VT, securing the patient with ICD and performing ablation of the arrhythmia [32]. It should be remembered that especially with structural damage to the ventricular muscle, ventricular disorders may recur. The antiarrhythmic therapy, and the selection of its intensity in relation to the effectiveness of the treatment and the patient’s clinical condition, require constant evaluation. It is important to avoid unnecessary medications (reducing proarrhythmic effects) and arrhythmogenic substances such as marijuana. Regular, moderate-intensity exercise, as well as preventive measures, including vaccination against influenza and COVID-19, play an important role in the treatment of heart disease. Finally, one should not forget about the practical aspects of treatment, such as the limitations related to the implantation of an ICD, which result in the impossibility of professional driving [33].

There has been a rapid growth in prescribing marijuana worldwide following the legalization of medicinal cannabis in different countries [34]. This increasing legalization gives the illusive impression that marijuana is rather safe, which also translates into its expanded recreational use. Since knowledge about marijuana’s serious side effects concerning circulatory system is not common, the physicians and other healthcare workers should have a low threshold for the possibility of marijuana being the underlying cause of adverse cardiovascular events [3].

## 4. Conclusions

Medical marijuana is a drug with potentially life-threatening side effects, and it affects the cardiovascular system in a number of ways even in young adults. Addressing marijuana-induced complications including ventricular arrhythmias is multidirectional, requiring a comprehensive approach to diagnosis and treatment that involves cardioverter-defibrillator implantation, source of arrhythmia ablation, pharmacotherapy and cannabis addiction therapy with strict cooperation with a psychiatrist.

## Data Availability

The study did not report any data.
